# Perioperative and Postoperative Complications of Ultrasound-Guided Totally Implantable Venous Access Ports via the Brachiocephalic Vein in Patients with Cancer: A Prospective Study

**DOI:** 10.7150/jca.55343

**Published:** 2021-01-01

**Authors:** Xingwei Sun, Xuming Bai, Yu Zhang, Liang Xu, Zepeng Yu, Yong Jin, Zhixiang Zhuang

**Affiliations:** 1 Department of Intervention, The Second Affiliated Hospital of Soochow University, Suzhou 215004, Jiangsu, China.; 2 Department of Orthopedics, The Second Affiliated Hospital of Soochow University, Suzhou 215004, Jiangsu, China.; 3 Department of General Surgery, Changshu Hospital Affiliated to Nanjing University of Chinese Medicine, Changshu, 215500, Jiangsu, China.; 4 Department of Oncology, The Second Affiliated Hospital of Soochow University, Suzhou 215004, Jiangsu, China.

**Keywords:** Totally implantable venous access port, brachiocephalic vein, ultrasound

## Abstract

**Objectives:** To evaluate the safety and efficacy of ultrasound (US)-guided totally implantable venous access ports (TIVAPs) via the right brachiocephalic vein (BCV) or the left BCV approach.

**Methods**: Patients requiring TIVAP for chemotherapy were included in the study. US-guided TIVAPs via BCV were used for patients from July 2018 to December 2018. General information about the patients (sex, age, and diagnosis), side (right or left), surgical procedures and complications were recorded.

**Results:** A total of 107 TIVAPs in 107 patients (ages 38-73 years) were included, 75 via the right BCV and 32 via the left BCV. All of the patients underwent successful surgery. The BCV was successfully punctured on the first attempt in 99 patients (92.52%). Two attempts were needed in 6 patients (5.61%), and three attempts were necessary in 2 patients (1.87%). The mean operation time was 29 ± 5 min (range: 24 to 38 min). No serious complications occurred during the surgery, except the formation of a local haematoma in 1 case after artery puncture. During the follow-up period of 12 months, the incidence of long-term complications was 3.74% (4/107), including 2 cases of catheter-related infection and 2 cases of fibrin sheath formation. No serious complications such as catheter malposition or rupture were found.

**Conclusion:** US-guided TIVAP via the BCV offers an alternative for adults with good needle guidance and a low rate of perioperative and postoperative complications.

## Introduction

In 1982, Niederhuber et al. placed the first totally implantable venous access port (TIVAP) via the cephalic vein using surgical techniques 1. It is considered one of the best achievements for cancer patients in the last 40 years, because it reduces the risk of chemotherapeutic drug infusions and significantly improves quality of life 2-4.

Due to the rapid development of ultrasound technology in recent years, US-guided TIVAP via the right brachiocephalic vein (BCV) was gradually used in adult cancer patients by clinicians, as reported in our previous studies 5-6. However, few prospective studies evaluated the safety and efficacy of this new approach. TIVAP via the left BCV was also considered a risk, and the left BCV approach was not included.

To evaluate the feasibility of the new approach for US-guided TIVAP via BVC (both right and left) in adult patients with cancer, a prospective study was performed in our centre. The perioperative complications, puncture success rate, operation time and postoperative complications were recorded and analysed.

## Methods

All patients agreed to participate in this clinical study, and the Ethics Committee approved our research protocol.US-guided TIVAPs via right or left BCV were used for adult patients with cancer from July 2018 to December 2018. All patients underwent complete preoperative examination, including routine blood tests, coagulation function, liver and kidney functions, and ultrasound evaluation of blood vessels, if necessary.

The exclusion criteria included abnormal clotting that could not be corrected, patients with neck cancer and local skin infection and inadequate visualization of the BCV using US.

### Materials

TIVAPs from Bard or B. Braun (BardPort, 8806061, 6F, 45 cm, Salt Lake City, UT, USA; B. Braun, 04436946, 6.5F, 45 cm, Ile-de-France, France) were used. A 6-12 MHz linear array US probe of a LOGIQ ultrasound device (General Electric, Fairfield, CT, USA) was used in all cases.

### Technique

The US probe was run down the internal jugular vein (IJV) to the supraclavicular region to obtain a longitudinal view of the BCV origin where the IJV and subclavian vein (SCV) join. The diameter and depth of the BCV and the presence of blood vessels, nerves and other tissues in the puncture route were evaluated.

The BCV was cannulated by advancing a needle under US guidance using the in-plane technique. No more than three puncture attempts were allowed during one approach.

### Implantation of TIVAP

Two trained senior interventional physicians performed the surgery in the operating theatre. Ultrasound-guided operation is a necessary skill and the trained interventional physicians were skilled in ultrasound-guided puncture. No sonographer involved.

The patient was in a supine position with the head turned 45 degrees to the side opposite the operative site.

**Figure 1:**
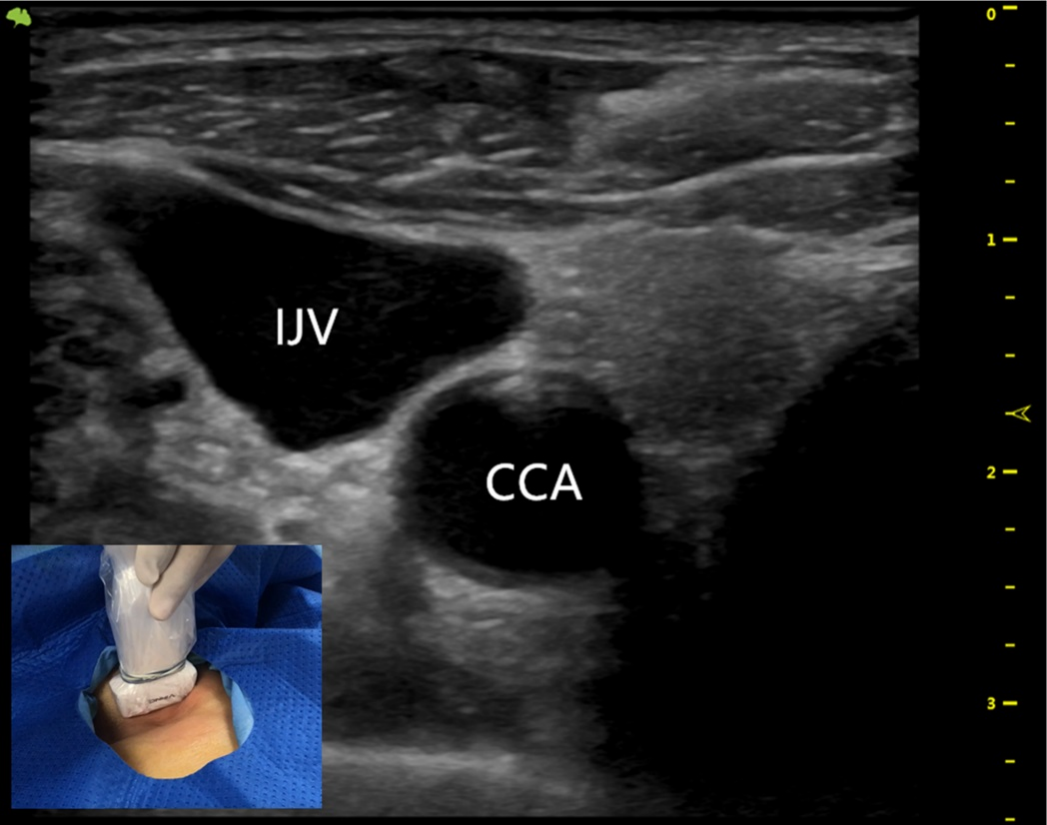
The ultrasound probe runs down the IJV showing the outboard IJV and the inboard CCA. IJV indicates the internal jugular vein; CCA indicates the common carotid artery.

**Figure 2:**
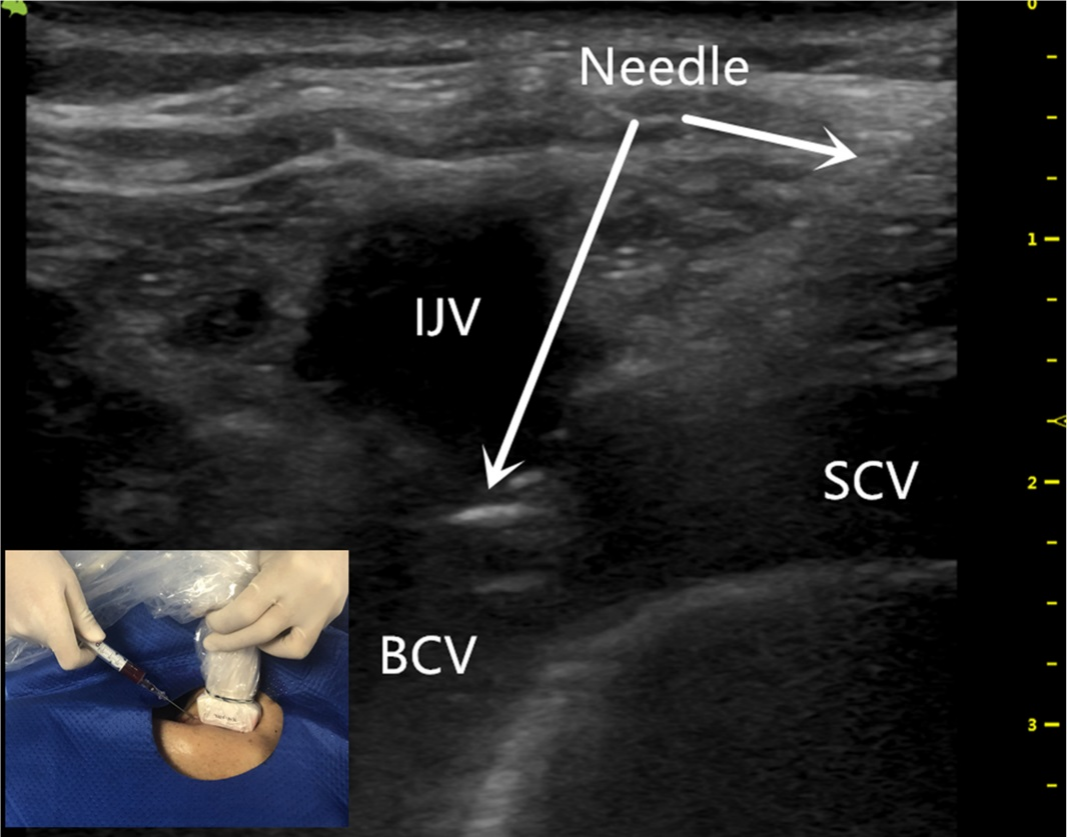
The ultrasound-guided successful puncture of the BCV needle insertion (white arrow) using the BCV longitudinal view, in-plane approach. BCV indicates the brachiocephalic vein; IJV indicates the internal jugular vein; SCV indicates the subclavian vein.

The US probe was run down the IJV (**Figure 1**) to the top of the sternoclavicular joint to obtain a longitudinal view of the BCV. The puncture site was locally anesthetized with 1% lidocaine. With the guidance of the US probe (the in-plane technique), the needle was advanced once the BCV was visualized on the US screen (**Figure 2**). After successful puncture, the guide wire, sheath, and catheter were entered sequentially.

The first attempt was defined as the first skin puncture. If three attempts to cannulate the BCV failed, the guide wire could not be successfully advanced or there was poor visualization of the BCV, the procedure was repeated on the opposite BCV.

**Figure 3:**
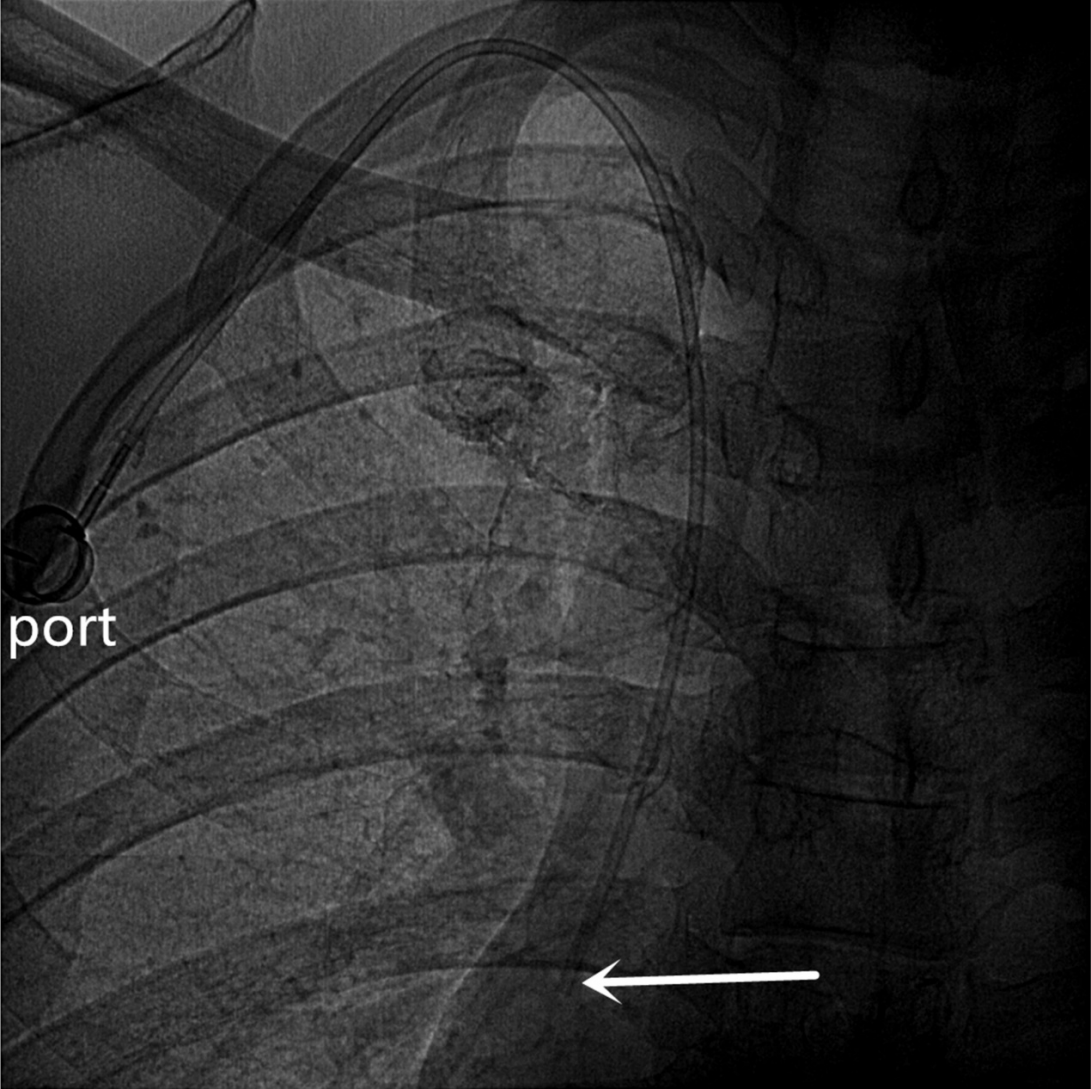
A totally implantable venous access port (TIVAP) is implanted via the right BCV approach, crossing over the right clavicle. The port is located on the right chest wall, and the tip of the catheter (white arrow) is located at the junction of the superior vena cava and the right atrium.

**Figure 4:**
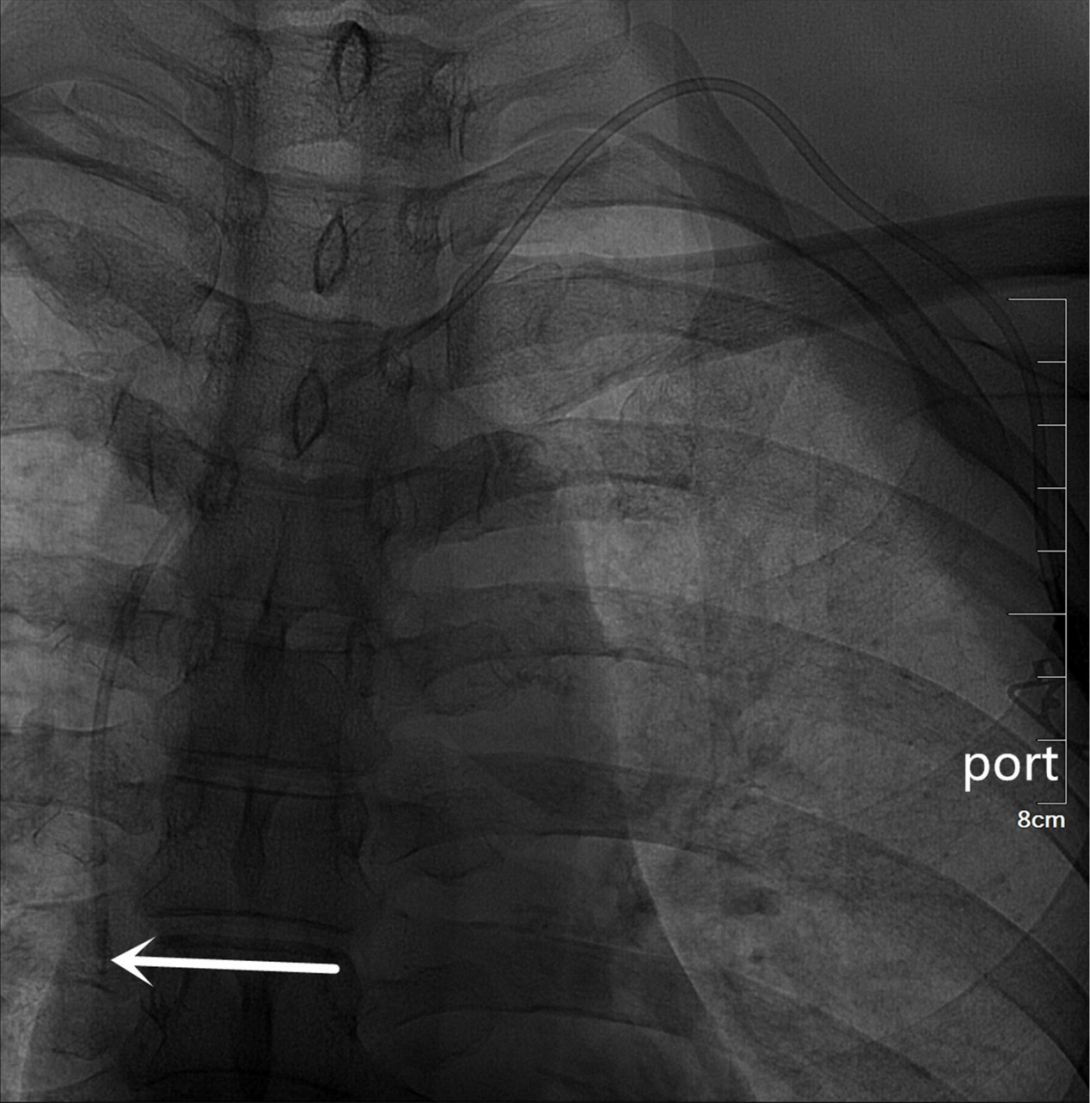
A totally implantable venous access port (TIVAP) is implanted via the left BCV approach, crossing over the left clavicle. The port is located on the left chest wall, and the tip of the catheter (white arrow) is located at the junction of the superior vena cava and the right atrium.

An artificial pocket of the appropriate size was created on the upper part of the chest wall, just enough to contain the port. A tunnel needle crossed over the supraclavicular region to connect the catheter and port in the pocket, and its tip was adjusted to the junction of the superior vena cava and the right atrium under fluoroscopy (**Figures 3 and 4**). Blood infusion and withdrawal were tested again to ensure that the infusion port functioned normally after the incision was closed.

### Maintenance of TIVAP

Specially trained nurses at the venous access care centre of our hospital maintained the TIVAPs. The catheter was flushed with 10 ml of 50~100 IU/ml heparin saline in a pulsed manner once every 4 weeks. If catheter dysfunction occurred, nurses promptly notified the interventional physician for examination or treatment. Regular maintenance is an important factor in a well-functioning infusion port.

### Data collection

General information of the patients (sex, age, diagnosis) and surgical procedures were recorded: side; catheter length; number of punctures; TIVAP brand; procedure time; perioperative complications, such as arterial puncture, pneumothorax, and local haematoma formation; postoperative complications, such as catheter-related infection, fibrin sheath formation, thrombosis, catheter malposition or rupture.

## Results

Overall, 107 patients were involved during a 4-month period, and the general information of patients is shown in **Table 1**. BCV was identified using US in all patients. All patients underwent successful surgery (100%), including 75 via the right BCV and 32 via the left BCV. The success rate for the first attempt was 92.52% (99/107). Two attempts were needed in 6 patients (5.61%), and three attempts were necessary in 2 patients (1.87%) (**Table 2**).

No serious complications occurred, except formation of a local haematoma in one case after artery puncture, which is shown in the right subclavian artery (SCA) using US (**Table 3**). After compression of the puncture site, the left BCV was used and the surgery was successful with one attempt.

The mean operation time was 29 ± 5 min (range: 24-38 min). The mean length of the implanted catheter was 20.4 ± 4.1 cm (range: 18-23 cm) in the right BCV approach and 25.0 ± 5.6 cm (range: 23-27 cm) in the left BCV approach (**Table 2**). The indwelling time was 269 ± 43 (range: 35-328) days. During the follow-up period of 12 months, the incidence of postoperative complications was 3.74% (4/107) (**Table 3**), including 2 cases of catheter-related infection and 2 cases of fibrin sheath formation. Due to the failure of active anti-infective and thrombolytic therapy, the four ports were removed unplanned.

No serious complications, such as catheter malposition or rupture, were found during the study.

## Discussion

The prospective study of a series of supraclavicular US-guided BCV for TIVAPs in adults demonstrated that the surgery was successful in all the patients. A successful puncture was obtained at the first attempt for most patients, and no serious complications occurred. The needle and BCV were clearly identified using this in-plane technique, and it offered a good view because the diameter was obviously increased after IJV and SCV confluence into the BCV.

The thoracic duct converges into the left BCV at the junction of the left IJV and the left SCV. The right BCV was preferable for the surgeons because the right BCV is straight, and the right BCV approach was performed to avoid thoracic duct damage as described in our previous studies 5-6.

However, other studies make us think twice. Beccaria studied, 78 adult patients who underwent central venous catheterization (CVC) via the left BCV, and no thoracic duct injury was found 7. The results of another study also showed that US-guided CVC via the left BCV was safe and effective in children 8. These studies suggest that TIVAP via the left BCV may not be taboo.

In contrast to previous studies, we included US-guided TIVAP via the left BCV approach in this study. The left BCV approach was preferred only when there were contraindications on the right operative site, e.g., right-side breast cancer or the requirement for radiation therapy.

The incidence of complications increased with the number of punctures 9, 10. Three attempts were needed in two patients in the present study. The BCV of one patient was severely distorted and slender, which increased the puncture difficulty. Another patient required more than two attempts due to the lack of puncture experience using this technique, and a local haematoma formed after artery puncture (shown as the right subclavian artery on US).

The arteries (SCA) are not compressible, which is disadvantage of the BCV approach compared to the IJV approach. Therefore, ultrasound guidance is necessary, and effective training reduces the occurrence of this complication. Preoperative coagulation examination is also very important, and coagulation function should be corrected before surgery if necessary 11. Once artery puncture occurred, the needle was withdrawn in time, the puncture site was compressed, and the opposite BCV was used. There were also reports of subclavian artery puncture with the SCV approach 12, 13, which is also not compressible, and is the same as the BCV approach.

Notably, the results showed no difficulty in advancing the guide wire in this study. There was no guide wire ectopy in the IJV or SCV during puncture, which may be due to an anatomical factor, i.e., the Y-shaped anatomical morphology of the IJV, SCV, and BCV.

The US identified the pleural fascia using the BCV approach in most patients. The puncture direction was parallel to the pleural fascia, which reduced the incidence of pneumothorax. By running down the IJV to the BCV origin, the brachiocephalic artery may be identified and easily excluded by the US probe to avoid artery puncture.

According to previous studies 14, 15, the cephalic venous approach using surgical techniques has a lower incidence of complications, and it is considered superior to the SCV approach. However, surgical techniques for TIVAP using the cephalic venous approach also have the disadvantages of a long operation time, a low success rate, and significant trauma 16, 17. The present study showed that US-guided TIVAP via the BCV approach resulted in a shorter surgical time and higher success rate.

Catheter-related infection was found in two cases 35 days and 127 days after surgery. Blood culture showed Staphylococcus aureus, and after the failure of intravenous antibiotic treatment, the TIVAPs were removed. The other two catheter dysfunctions were found 82 days and 93 days after the surgery, DSA imaging showed fibrin sheath formation, they also led to port withdrawal after failure of active thrombolytic therapy.

Catheter malposition or rupture was not found during the study. The exact mechanism of the low incidence of catheter malposition or rupture is not clear. In a retrospective study of 280 patients with TIVAP via IJV, the incidence of perioperative complications and long-term complications in the right IJV group were 1.43% (4/280) and 3.93% (11/280), respectively, including catheter malposition in 2 cases, and catheter fracture in 1 case 18.

Compared to the IJV or SCV approach, the BCV approach has the advantages of low mobility, smooth catheter shape and avoidance of pinch-off syndrome (POS) with supraclavicular approach. These factors greatly reduce the probability of catheter malposition or catheter rupture 19-21. The specific Y-shaped anatomical structure formed by the IJV, SCV and BCV also plays an important role, and the Y-shaped anatomical structure makes it difficult for the catheter in the BCV to enter the IJV or SCV.

Because that the preliminary results of the cases are limited and there were no comparisons with other puncture approaches (IJV, SCV), there is a clear need for larger sample clinical trials to confirm the advantages of this BCV approach technique. The incidence of postoperative complications due to some unavoidable reasons may not be accurate in our study, because we may have missed some TIVAPs that were inserted in our hospital but were taken out in other medical institutions.

In conclusion, with a good view of the needle and the BCV, US-guided TIVAP via the BCV offers a new approach for adult patients with cancer. The success rate may be improved with the inclusion of more patients included, but it may also result in more complications. This study provide evidence for the use of the BCV approach as an alternative choice for clinicians when TIVAPs are required by patients.

## Figures and Tables

**Table 1:** Patients' characteristics (N=107)

Characteristics	N. (%)
Female/Male	69/38
Age (years) (Mean ± SD)	51.5±15.8 (38-73)
Hepatocellular Carcinoma	36
Lung Cancer	25
Right/Left breast cancer N (%)	20/17
Colorectal Carcinoma	9

**Table 2:** Details of US-guided TIVAPs via BCV (N=107)

Details	N. (%)
Success rate of surgery (%)	107 (100)
TIVAP via the right BCV (%)	75 (70.09)
TIVAP via the left BCV (%)	32 (29.91)
Success rate of first attempt (%)	99 (92.52)
Two attempts were needed	6 (5.61)
Three attempts were needed	2 (1.87)
Operation time (minutes) (Mean ± SD)	29 ± 5 (24-38)
Length of the right catheter introduction (cm) (Mean ± SD)	20.4 ± 4.1 (18-23)
Length of the left catheter introduction (cm) (Mean ± SD)	25.0 ± 5.6 (23-27)
TIVAPs time (days)	269 ± 43 (35-328)

**Table 3:** Complications and actions taken (N=107)

Complications	No. (%)	Actions taken and outcome
Artery perforated	1(0.93)	Press the puncture site,Self-limited
Catheter-related infection	2(1.87)	Antibiotics and port removal
Fibrin formation	2(1.87)	Thrombolysis and port removal
Total	(4.67)	
